# Citric acid assisted phytoextraction of nickle from soil helps to tolerate oxidative stress and expression profile of *NRAMP* genes in sunflower at different growth stages

**DOI:** 10.3389/fpls.2022.1072671

**Published:** 2022-12-01

**Authors:** Munazza Ijaz, Mahmood-ur-Rahman Ansari, Hayat Ali Alafari, Muhammad Iqbal, Dalal S. Alshaya, Sajid Fiaz, Hafiz Muhammad Ahmad, Muhammad Zubair, Pia Muhammad Adnan Ramzani, Javed Iqbal, Asmaa M. Abushady, Kotb Attia

**Affiliations:** ^1^ Department of Bioinformatics and Biotechnology, Government College University, Faisalabad, Pakistan; ^2^ Department of Biology, College of science, Princess Nourah bint Abdulrahman University, Riyadh, Saudi Arabia; ^3^ Department of Environmental Science and Engineering, Government College University, Faisalabad, Pakistan; ^4^ Department of Plant Breeding and Genetics, The University of Haripur, Haripur, Pakistan; ^5^ Cholistan Institute of Desert Studies, The Islamia University, Bahawalpur, Pakistan; ^6^ Department of Agricultural Engineering, Khwaja Fareed university of Engineering and Information Technology, Rahim Yar Khan, Pakistan; ^7^ Biotechnology School, Nile University, Sheikh Zayed, Giza, Egypt; ^8^ Department of Genetics, Agriculture College, Ain Shams University, Cairo, Egypt; ^9^ Center of Excellence in Biotechnology Research, King Saud University, Riyadh, Saudi Arabia; ^10^ Rice Biotechnology Lab, Rice Department, Field Crops Research Institute, ARC, Sakha, Egypt

**Keywords:** soil pollution, toxicity, gene expression, proline, citric acid, nickel

## Abstract

**Introduction:**

Soil polluted with Nickel (Ni) adversely affects sunflower growth resulting in reduced yield. Counterbalancing Ni toxicity requires complex molecular, biochemical, and physiological mechanisms at the cellular, tissue, and whole plant levels, which might improve crop productivity. One of the primary adaptations to tolerate Ni toxicity is the enhanced production of antioxidant enzymes and the elevated expression of Ni responsive genes.

**Methods:**

In this study, biochemical parameters, production of ROS, antioxidants regulation, and expression of *NRAMP* metal transporter genes were studied under Ni stress in sunflower. There were four soil Ni treatments (0, 50, 100, and 200 mg kg^-1^ soil), while citric acid (CA, 5 mM kg^-1^ soil) was applied on the 28^th^ and 58^th^ days of plant growth. The samples for all analyses were obtained on the 30^th^ and 60^th^ day of plant growth, respectively.

**Results and discussion:**

The results indicated that the concentrations of Ni in roots and shoots were increased with increasing concentrations of Ni at both time intervals. Proline contents, ascorbic acid, protein, and total phenolics were reduced under Ni-stress, but with the application of CA, improvement was witnessed in their contents. The levels of malondialdehyde and hydrogen peroxide were enhanced with the increasing concentration of Ni, and after applying CA, they were reduced. The contents of antioxidants, i.e., catalase, peroxidase, superoxide dismutase, ascorbate peroxidase, dehydroascorbate reductase, and glutathione reductase, were increased at 50 ppm Ni concentration and decreased at higher concentrations of Ni. The application of CA significantly improved antioxidants at all concentrations of Ni. The enhanced expression of *NRAMP1* (4, 51 and 81 folds) and *NRAMP3* (1.05, 4 and 6 folds) was found at 50, 100 and 200ppm Ni-stress, respectively in 30 days old plants and the same pattern of expression was recorded in 60 days old plants. CA further enhanced the expression at both developmental stages.

**Conclusion:**

In conclusion, CA enhances Ni phytoextraction efficiency as well as protect plant against oxidative stress caused by Ni in sunflower.

## 1 Introduction

Rapid urbanization has caused severe environmental issues like a high accumulation of heavy metals (HMs) in soil, which is the main reason for soil pollution (Shahbaz et al., 2018; Turan et al., 2018). The HMs pollution results in halted growth, decreased yield, and negatively affects plant development (Sevik et al., 2018; Rai et al., 2019; Yakamercan et al., 2021). Soil pollution with Nickel (Ni) is a worldwide concern (Biswas et al., 2017). It is released into the soil by both anthropogenic and natural sources (Pujari and Kapoor, 2021). The anthropogenic sources of its contamination are waste originating from electroplating industries (Lee et al., 2017), Ni and steel amalgams, Ni and iron amalgams (Harasim and Filipek, 2015), mining and smelting of Ni ores, wastewater, pesticides, fertilizers, sewage sludge and Ni-Cd batteries (Chakankar et al., 2017). The natural sources of Ni contamination are volcanic eruptions and weathering of igneous rocks (Shahbaz et al., 2018). The main symptoms of plants under Ni stress are necrosis, chlorosis, inhibition of enzymatic activities, and stunted growth of roots. Nickel toxicity also disturbs several physiological responses, including respiration, water-plant relation, transport of assimilates, mineral nutrition, and photosynthesis (Gajewska et al., 2009; Maharajan et al., 2022). When polluted with Ni, soil fertility and health (Ngole-jeme and Fantke, 2017) are adversely affected which may affect plants growth and development (Soares et al., 2016). Thus, eco-friendly remedial actions are needed to decontaminate the soil from Ni. Phytoextraction is an eco-friendly technique involving hyperaccumulator plants that can remove Ni from Ni-contaminated soils without harming the environment. High biomass production, fast growth rate, long roots, high translocation factor, and good accumulation efficiency are the desired characteristics of hyperaccumulator plants (Song et al., 2015; Awa and Hadibarata, 2020; Antonkiewicz et al., 2022). As a result, these plants can accumulate high concentrations of HMs in their parts without showing any toxic effects (Rascio and Navari-Izzo, 2011; Nedjimi, 2021).

*Helianthus* species have been reported to show tolerance against various HMs, hydrocarbons, and other contaminants. Helianthus is a hyper-accumulator plant due to its fast growth rate, accumulates HMs, and high biomass production (Chauhan and Mathur, 2018). A plant’s hyperaccumulation potential is increased by enhancing the bioavailability of HMs in soil and could be improved by applying chelating agents. These chelating agents are synthetic like EDTA as well as organic like citric acid (CA), maleic acid (MA), etc. (Chigbo and Batty, 2013). Synthetic chelators are useful for phytoextraction, but they are non-biodegradable and contaminate groundwater (Anwer et al., 2012). Different organic acids, including CA, have fewer leaching hazards, and they are biodegradable, so they are preferred to be used (Farid et al., 2017). CA is quite beneficial for the uptake of nutrients and HMs from the soil *via* plants (Vagner et al., 2013). The smaller amount of CA can increase the uptake of HMs by plants, while a higher concentration may cause strong phytotoxic reactions in some plant species (Turgut et al., 2004). The CA is proven beneficial for mobilizing Ni in the soil and reduces the effects of heavy metal stress by initiating the production of antioxidants. When plants suffer from Ni stress, a large number of reactive oxygen species (ROS) like malonaldehyde (MDA) and hydrogen peroxide (H_2_O_2_) are produced in the plants that can cause cellular damage and eventually result in oxidative stress (Sharma et al., 2012). The plants have an antioxidative defense mechanism that helps plants fight against ROS. Various antioxidants like superoxide dismutase (SOD), peroxidase (POD), ascorbate peroxidase (APX), dehydroascorbate reductase (DHAR), glutathione reductase (GR), and catalase (CAT) have been identified to participate in the HMs tolerance mechanism in hyper-accumulators plants (Zhao et al., 2012).

The expression of HMs tolerant genes in hyper-accumulator plants is also the main factor responsible for stress tolerance (Mishra et al., 2017). The molecular studies have identified genes accountable for chelation, cell wall modification, root to shoot translocation and the production of antioxidants (Hanikenne et al., 2008; Zhang and Evans, 2013; Peng et al., 2017). Numerous transmembrane transporters like *NRAMPs* (natural resistance-associated macrophage proteins), *HMA* (heavy metal ATPase), YSL (yellow strip-like), CDF (cation diffusion facilitators), MTP (metal tolerance protein), and ZIP (ZRT, IRT-like proteins) are responsible for uptake of HMs in plants (Singh et al., 2019a; Maharajan et al., 2022). In all eukaryotes, membrane intrinsic metal transporters with substrate preferences for many metals, including Ni and Fe, are *NRAMP* transporters (Nakanishi-Masuno et al., 2018). Various analyses of higher plants showed that the rice and *Arabidopsis* genomes have seven and six genes that encode *NRAMP* transporter proteins (Singh et al., 2019a). They are responsible for transporting different metal cations, such as Ni^+2^, Mn^+2^, Al^+3^, Cd^+2,^ and Zn^+2^. For example, uptake of Mn or Fe is regulated by *NRAMP1* in *Arabidopsis* that is localized in the plasma membrane (Cailliatte et al., 2010; Lanquar et al., 2010). The Al^+3^ tolerance is achieved by the expression of *NRAMP4* in rice (Li et al., 2014). The Fe, Mn, and Cd uptake is regulated by the expression of *OsNRAMP5* in roots (Ishimaru et al., 2012; Sasaki et al., 2012). Ni stress is one of the significant threats to animal and plant health. Higher Ni concentrations results in significant yield losses, however adaptive mechanisms for Ni tolerance are not yet well illustrated. Therefore, present research was designed to understand the mechanism of oxidative stress caused by Ni. The CA treatments were applied to enhance Ni uptake in plants.

## 2 Materials and methods

### 2.1 Plant experiment

The seeds of Pioneer Hybrid 6946 sunflower (*Helianthus annus*) were purchased from Ayub Agricultural Research Institute, Faisalabad, Pakistan. After surface sterilization with 70% ethanol, the seeds were soaked in distilled water and kept in the dark for 48 hours to initiate germination. The six seeds were sown in pots having 10 kg of soil and placed in the botanical garden of Government College University, Faisalabad. Before sowing seeds in the pots, the soil was spiked with Ni in four variable concentrations (0, 50, 100, and 200 mg kg^-1^ soil) (**Table 1**). All treatments were performed in three replicates. After the seeds were germinated, one healthy plant per pot was maintained. After 28 and 58 days of growth, the plants were treated with CA (5 mM kg^-1^ soil) separately and in combinations with all concentrations of Ni. After two days of each CA treatment, the samples from roots and shoots were taken and transported to the laboratory for biochemical and molecular analyses. The plants grown in 0 ppm concentration of Ni were used as control. The experiment was conducted in triplicate using a completely randomized design (CRD).

**Table 1:** Treatments performed in this pot experiment.

Treatments	Ni (mg kg^-1^ soil)	Citric acid (mM kg^-1^ soil)
Control	−	−
Ni 50	50	−
Ni 100	100	−
Ni 200	200	−
CA	−	5
Ni 50 + CA	50	5
Ni 100 + CA	100	5
Ni 200 + CA	200	5

### 2.2 Ni distribution in plant parts and bioavailable Ni fraction in soil

The 0.1 g of dried samples (composite sample from three plants from each replicate) of roots and shoots were ground and digested with a mixture of acids (5: 1, v/v, HNO_3_: HClO_4_) (Jones and Case, 1990). Likewise, DTPA extractable Ni was determined by following the method developed by Lindsay and Norvell (1978). The concentrations of Ni in plant digest and DTPA extract were measured on ICP-MS (PerkinElmer’s NexION^®^ 2000).

### 2.3 Biochemical compounds in leaves

#### 2.3.1 Proline

The Proline content was determined spectrophotometrically according to the procedure developed by Bates et al. (1973). The sulfosalicylic acid (3%) was applied to homogenize leaves, and then samples were centrifuged at 11,500×g. Next, glacial acetic acid and acid ninhydrin were mixed with the supernatant, followed by 1 h incubation at 100°C. The toluene was added after cooling the mixture, and the chromophore containing toluene was analyzed spectrophotometrically at 520 nm. The proline content in the sample was measured by making a comparison with the standard curve of known concentrations.

#### 2.3.2 Ascorbic acid

Ascorbic acid (AsA) contents were measured using approximately 0.5g of fresh leaves properly mixed in 5% meta-phosphoric acid with 1mM EDTA and centrifuged for 12 minutes at 4°C on 11,500×g. Further, the supernatant was separated for analysis of AsA. Then, incubation was given with 0.1M dithiothreitol at room temperature for 1 h to reduce the oxidized fraction. The absorbance was measured at 265nm, and 1 unit of ascorbate oxidase (AO) was used. Finally, the calculation of oxidized ascorbate ((DHA) (DHA= reduced AsA – total AsA) was done (Hasanuzzaman et al., 2011; Nahar et al., 2016a).

#### 2.3.3 Total protein contents

Total protein contents were measured by Bradford protein assay, and bovine serum albumin (BSA) was used as a standard (Bradford, 1976).

#### 2.3.4 Total phenolic contents

The total phenolic content in leaf extracts was determined colorimetrically (Singleton et al., 1999). The absorbance of samples with gallic acid standards was taken at 760nm.

#### 2.3.5 Chlorophyll contents

The chlorophyll contents (both a and b) were determined according to the standard procedure (Arnon, 1949). Ten (10) mL of 80% v/v acetone was applied to leaves (0.5 g approximately), and obtained supernatant was centrifuged (10 min at 2000×g) followed by dilution of supernatant. A UV-visible spectrophotometer was used to measure the absorbance at 663 nm and 645 nm to measure the content of chlorophyll a (Chl a) and chlorophyll b (Chl b), respectively.

### 2.4 Plant stress and antioxidant enzymes

#### 2.4.1 Contents of MDA and H_2_O_2_

The thiobarbituric acid (TBA) assay was used to determine leaf MDA content (Cakmak and Marschner, 1992). The fresh plant leaves (0.2g) were homogenized in a solution of TBA (0.5%) in 20% of trichloroacetic acid (TCA). Then, the mixture was heated at 95°C for 30 min. Additionally, samples were cooled and centrifuged for 5 min at 3000×g. The absorbance was determined from supernatant at 532 nm and 600 nm. The formula for calculating specific absorbance of MDA is: specific absorbance of MDA = absorbance at 600 nm - absorbance at 532 nm. The H_2_O_2_ content in sunflower leaves was measured by the method defined by Veljovic-jovanovic et al. (2002). The 0.1g of fresh leaves were collected, immediately stored in liquid nitrogen, and analyzed. To remove phenolic compounds, samples were mixed with 1M HCLO_4_ (1.5 mL) and insoluble polyvinylpyrrolidone (0.1 g). The mixtures were centrifuged in a temperature control centrifuge for 10 mins at 4°C. The H_2_O_2_ content from the supernatant was calculated by Cheeseman (2006) method.

#### 2.4.2 Activities of antioxidant enzymes

The 500 mg leaf tissues were mixed in ice-cold 50 mM K-P buffer (1mL; pH = 7.0) having 100 mM potassium chloride, β-mercaptoethanol (5mM), glycerol (10% w/v), and ascorbate (1 mM) by a pre-cooled pestle and mortar. The mixtures were centrifuged for 10 mins at 11,500×g in a refrigerated centrifuge machine, and enzyme activities were measured by supernatant. The activity of CAT was measured by the method of Hasanuzzaman et al. (2011) by determining the decline in absorbance at 240nm. The enzyme extract was added with the homogenate having a K-P buffer of neutral pH and H_2_O_2_. The enzyme activity was measured through extinction coefficient 39.4 M^-1^ cm^-1^. SOD and POD activities were estimated through the xanthine oxidase method by El-shabrawi and Kumar (2010). The homogenate has enzyme extract, K-P buffer, xanthine, xanthine oxidase, catalase, and nitroblue tetrazolium chloride (NBT). The change in absorbance was measured at 560 nm, and enzyme activities were expressed by units (quantity of enzyme needed to restrict 50% NBT reduction) mg^-1^ protein. Similarly, the APX activity was calculated by the procedure of Nakano and Asada (1981) with a mixture of enzyme extract, EDTA, H_2_O_2_, AsA, and K-P buffer. The APX activity was determined by estimating the decrease in absorbance at 290 nm, and the extinction coefficient of 2.8 mM^-1^ cm^-1^ was used to calculate the enzyme activity. Likewise, DHAR activity was measured by the method of Nakano and Asada (1981) with the reaction mixture containing dehydroascorbate (DHA), EDTA, K-P buffer, glutathione (GSH), and enzyme extract. The activity was calculated after monitoring the variations in absorbance at 265 nm *via* 14mM^-1^cm^-1^ as extinction coefficient. The method of Hasanuzzaman et al. (2011) was opted to measure the GR activity *via* observing the change in the absorbance at 340 nm. The reaction mixture was composed of EDTA, NADPH, glutathione disulfide (GSSG), enzyme extract, and K-P buffer, and extinction coefficient of 6.2 mM^−1^ cm^−1^ was applied.

### 2.5 Isolation of RNA and analysis of gene expression

RNA was isolated from leaves obtained from the plant on 30 and 60 days of growth using the Trizol method (Thermo Scientific, USA). The RNA was analyzed quantitatively and qualitatively using the NanoDrop Spectrophotometer (Colibri Microvolume Spectrometer, Titertek-Berthold, Germany) and agarose gel electrophoresis (1%), respectively. The isolated RNA was treated with DNase free from RNase (Thermo Scientific, USA) to avoid contamination of DNA. The first-strand cDNA was synthesized by Reverse Transcriptase Polymerase Chain Reaction (RT-PCR) using 1 µg of purified RNA as a template. Gene expression levels were studied by quantitative real-time PCR using SYBER Green qPCR Master Mix (ThermoFisher Scientific, USA) in CFX96 Real-Time PCR System (BIO-RAD, USA). The variations in gene expression were calculated using the 2^−ΔΔCt^ analysis method. The quantification was carried out by the *Actin* gene as the reference gene. The specific primers for *NRAMP* genes used in qPCR can be seen in **Table 2**.

**Table 2:** List of primers used to study the expression of *NRAMP* genes through real-time PCR.

S. No.	Primer type and name	Sequence (5′-3′)
**1**	*NRAMP1*-Forward	CGGTGTTCTTCTGACAGG
	*NRAMP1*-Reverse	TCCGAGCAAAGAGATTGC
**2**	*NRAMP3*- Forward	ACAGTTCATAATGGGCGG
	*NRAMP3*- Reverse	AAGCACGTTAAGCCACTC
**3**	*Actin*- Forward	TCATGAAGATCCTGACGGAG
	*Actin*- Reverse	AACAGCTCCTCTTGGCTTAG

### 2.6 Quality assurance and quality control

Certified reference materials (CTA-OTL-1 for plant and DCI 7004 for soil analysis) and blank samples were used to ensure quality control. Recoveries for Ni were in the ranges of 94−97% (plant reference material) and 94−98% (soil reference material), respectively. All glassware and consumable items used in the digestion and extraction were initially soaked in diluted HNO_3_ (12 _h_) and later rinsed with deionized water several times.

### 2.7 Statistical analysis

The experiment was conducted in a completely randomized design with three replication. The average value for each parameter was calculated by Microsoft Excel 2013. A one-way analysis of variance (ANOVA) in Statistix 8.1 (Analytical Software, Tallahassee, USA) was used to interpret the results. For the determination of significant variance amongst treatment means (P < 0.05), the least significant difference (LSD) test was performed (Steel et al., 1997).

## 3 Results

### 3.1 Ni distribution in plant parts and bioavailable Ni fraction in soil

**Figure 1:**
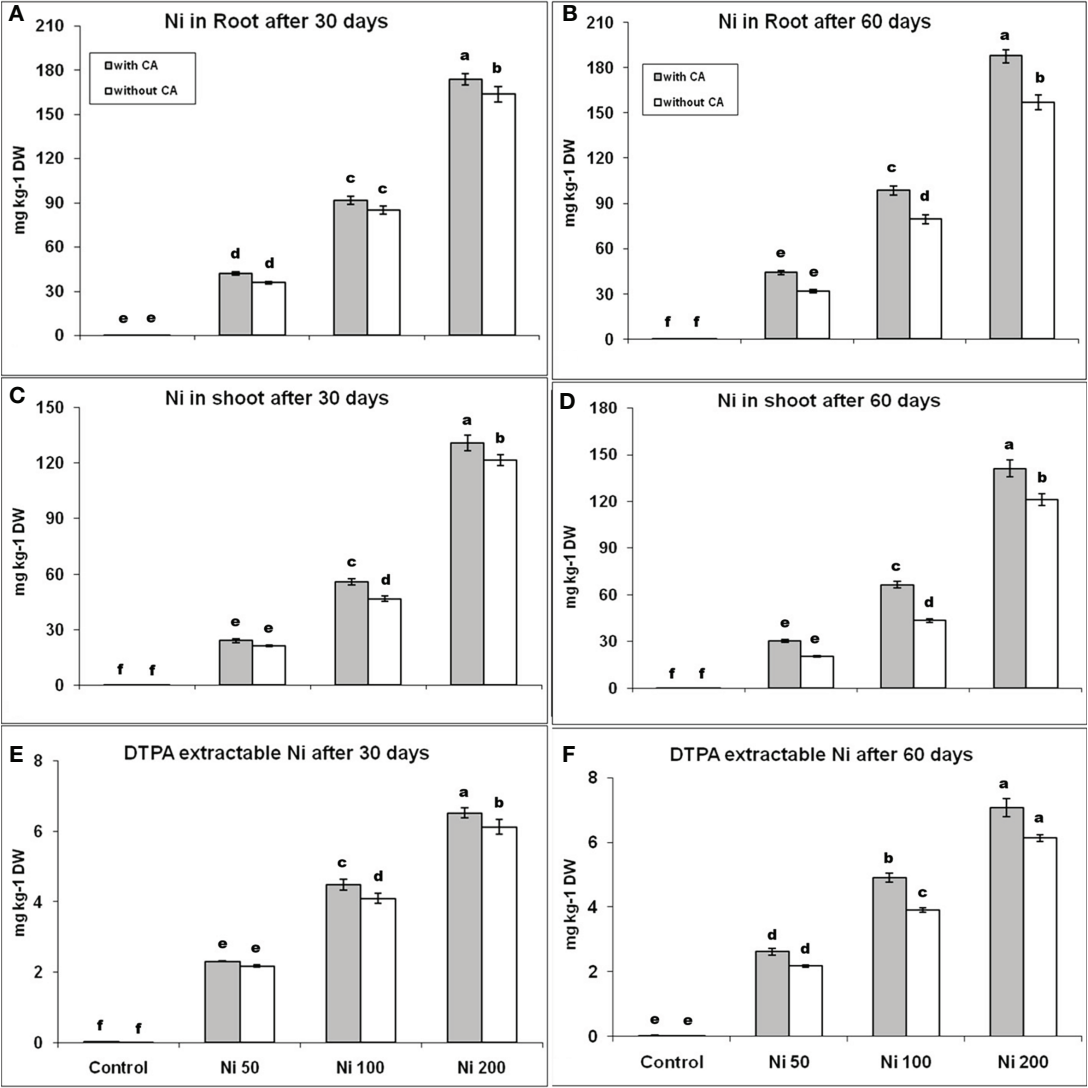
Root Ni content at 30th day **(A)**, and at 60th day **(B)**, shoot Ni content at 30th day **(C)**, and at 60th day **(D)**, DTPA extractable Ni at 30th day **(E)**, and at 60th day **(F)**. The bars designated with Ni 50, Ni 100, and Ni 200 represent Ni concentration at 50, 100, and 200ppm, respectively. “With CA” means these Ni-stressed plants are supplemented with 5 mM CA and vice versa for “Without CA”. The bars denoted by different letters are significantly variable at P≤ 0.05 using the LSD test.

The plants accumulated Ni dose-dependent in their roots and shoots, while high Ni contents were observed in roots compared to shoots (**Figures 1A–D**). The Ni uptake was increased with the addition of CA in both roots and shoots after 30 and 60 days. The Ni absorption by roots was enhanced by 17%, 8%, and 6%, as well as the level of Ni in shoots, was boosted by 13%, 20%, and 8% in 50, 100, and 200 ppm Ni-stressed plants supplemented with CA, respectively, in comparison with only Ni-stressed plants at 30^th^ day. The percentage difference in Ni content, in both roots and shoots, between plants bearing only stress and stress with CA was increased on the 60^th^ day. The DTPA extractable Ni content was raised by 6%, 10%, and 7% on the 30^th^ day, while 21%, 25%, and 15% on the 60^th^ day, in the plant having 50, 100, and 200 ppm Ni and augmented with CA, respectively, in contrast to only Ni-stressed plants (**Figures 1E, F**). The contents of DTPA extractable Ni were increased with CA supplementation on Ni stressed plants.

### 3.2 Biochemical compounds

**Figure 2:**
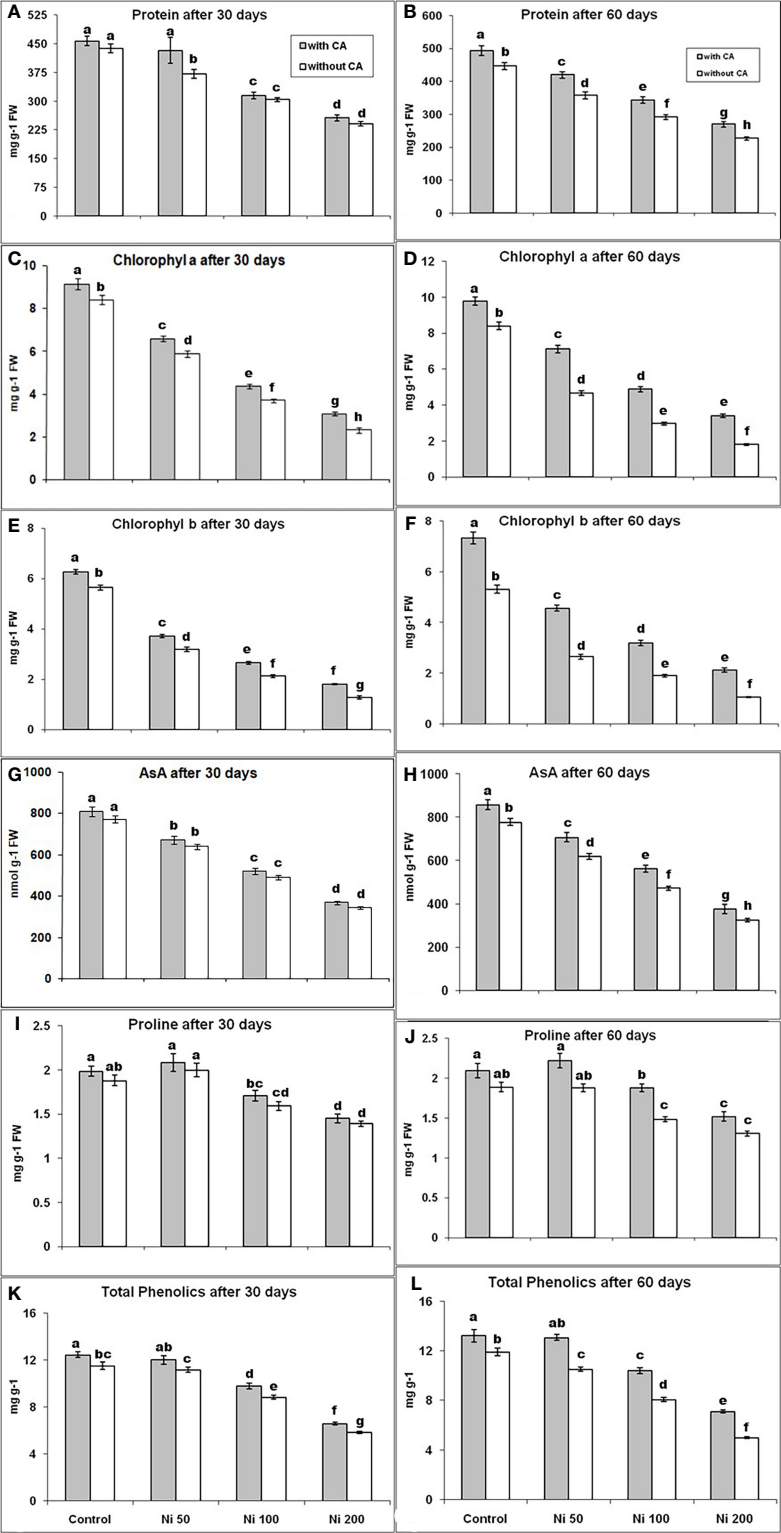
Protein content at 30^th^ day **(A)**, Protein content at 60^th^ day **(B)**, Chl a content at 30^th^ day **(C)**, Chl a content at 60^th^ day **(D)**, Chl b content at 30^th^ day **(E)**, Chl b content at 60^th^ day **(F)**, AsA content at 30^th^ day **(G)**, AsA content at 60^th^ day **(H)**, Proline content at 30^th^ day **(I)**, Proline content at 60^th^ day **(J)**, Total phenolics content at 30^th^ day **(K)** and Total phenolics content at 60^th^ day **(L)**, in sunflower plants. The bars designated with Ni 50, Ni 100, and Ni 200 represent Ni concentration at 50, 100, and 200ppm, respectively. “With CA” means these Ni-stressed plants are supplemented with 5 mM CA and vice versa for “Without CA”. The bars denoted by different letters are significantly variable at P≤ 0.05 using the LSD test.

The proline content was elevated under 50ppm stress of Ni by 6% and 11% on the 30^th^ day, with and without CA application, respectively, in contrast with control. However, it was decreased by 15% and 26% under 100 and 200 ppm Ni-stressed plants of 30 days, respectively, compared to control (**Figures 2G, H**). The 100 and 200 ppm Ni-stressed plants augmented with CA showed a lower decrease in protein contents on the 30^th^ day (**Figure 2A**). Effects of Ni-stress on protein contents were minimized with the supplementation of CA in 60 day old plants (**Figure 2B**). The content of AsA had shown a declined behavior with the increasing concentration of Ni at both time intervals (**Figures 2C, D**). A similar trend for proline as well as total phenolics was observed (**Figures 2I–L**). The Chl a concentration was reduced by 30% and 44% under 50ppm Ni-stress; declined by 56% and 65% during 100ppm Ni stress and decreased by 72% and 79% in 200ppm Ni-stress, while, Chl b content was reduced by 43% and 50% during 50 ppm Ni-stress; reduced by 62% and 64% in 100 ppm Ni-stress and declined by 77% and 80% under 200 ppm Ni-stress, at 30^th^ and 60^th^ day, respectively, as compared to control. The contents of Chl (a, b) were decreased abruptly with increasing the stress level on the 30^th^ and 60^th^ day, but their levels were observed better after the application of CA (**Figures 2C–F**).

### 3.3 Oxidative stress indicators

**Figure 3:**
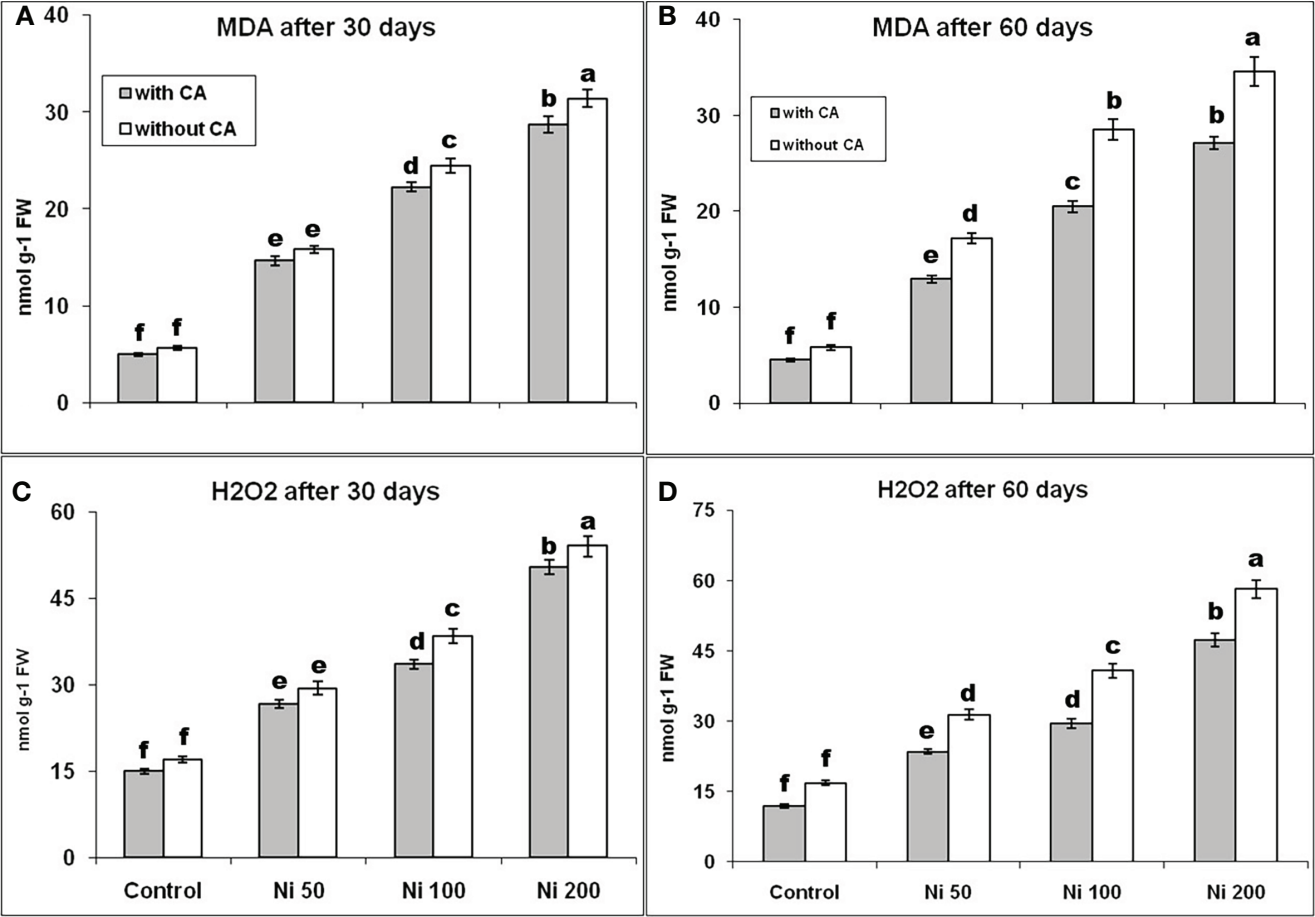
MDA content at 30^th^ day **(A)**, MDA content at 60^th^ day **(B)**, H_2_O_2_ content at 30^th^ day **(C)**, and H_2_O_2_ content at 60^th^ day **(D)** in sunflower plants. The bars designated with Ni 50, Ni 100, and Ni 200 represent Ni concentration at 50, 100, and 200ppm, respectively. “With CA” means these Ni-stressed plants are supplemented with 5 mM CA and vice versa for “Without CA”. The bars denoted by different letters are significantly variable at P≤ 0.05 using the LSD test.

The MDA concentration on the 30^th^ day was enhanced by 179%, 332%, and 445%, and on the 60^th^ day was elevated by 197%, 393%, and 497% under Ni-stress of 50, 100, and 200ppm, respectively, as compared to control. The MDA content declined with the application of CA at both time intervals (**Figures 3A, B**). A similar trend had been observed in the variation of H_2_O_2_ content (**Figures 3C, D**).

### 3.4 Antioxidant enzymes

**Figure 4:**
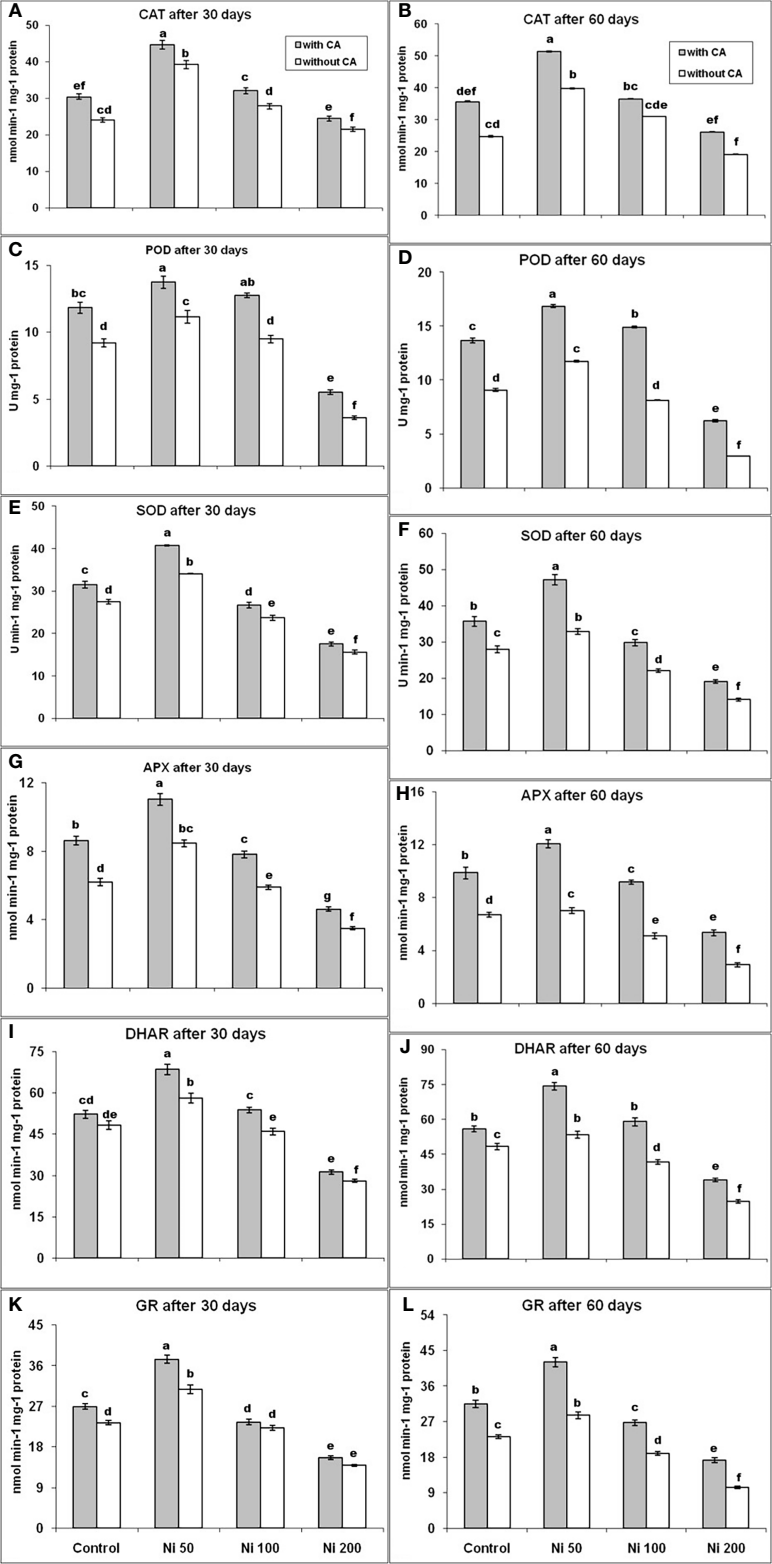
CAT after 30 days **(A)**, CAT after 60 days **(B)**, POD after 30 days **(C)**, POD after 60 days **(D)**, SOD after 30 days **(E)**, SOD after 60 days **(F)**, APX after 30 days **(G)**, APX after 60 days **(H)**, DHAR after 30 days **(I)**, DHAR after 60 days **(J)**, GR after 30 days **(K)** and GR after 60 days **(L)**, in sunflower plants. The bars designated with Ni 50, Ni 100, and Ni 200 represent Ni concentration at 50, 100, and 200ppm, respectively. “With CA” means these Ni-stressed plants are supplemented with 5 mM CA and vice versa for “Without CA”. The bars denoted by different letters are significantly variable at P≤ 0.05 using the LSD test.

Compared to control, 50 and 100 ppm Ni stress had a positive effect on the production of CAT, and it was increased by 63% and 16% on the 30^th^ day, while elevated by 61% and 25% after 60 days, respectively. However, 200 ppm Ni-stress decreased the production of CAT by 11% on the 30^th^ day and 23% on the 60^th^ day, in contrast to control. The addition of CA to Ni-affected plants had increased the content of CAT in comparison to control by 85%, 33%, and 1% at 30-days old plants, whereas it elevated by 107%, 48%, and 6% in 60-days old plants, under 50, 100 and 200ppm Ni-stress, respectively (**Figures 4A, B** **)**. The content of POD had behaved the same as CAT production except under 200 ppm Ni-stress, where even after the application of CA could not restore the production of POD (**Figures 4C, D**). The SOD and APX production had shown a similar trend. The SOD and APX levels were increased by 24% and 37%, and the application of CA enhanced their production 48% and 78% under 50ppm Ni-stress, while their concentration was decreased by 14% and 5% under 100ppm Ni-stress and elevated by 3% and 26% in 100ppm Ni-stressed plants supplemented with CA at the 30^th^ day, respectively, in comparison to control. Under 200ppm Ni-stress, both SOD and APX concentrations were reduced by 43%, compared to control, in 30 days old sunflower plants. In 60-days old plants, similar behavior of SOD and APX was observed. The application of CA in Ni-stressed plants improved the production of SOD and APX on the 30th day and the 60^th^ day (**Figures 4E–H**). The DHAR and GR followed the same trend as SOD and APX (**Figures 4I–L**).

### 3.5 Gene expression profiling

**Figure 5:**
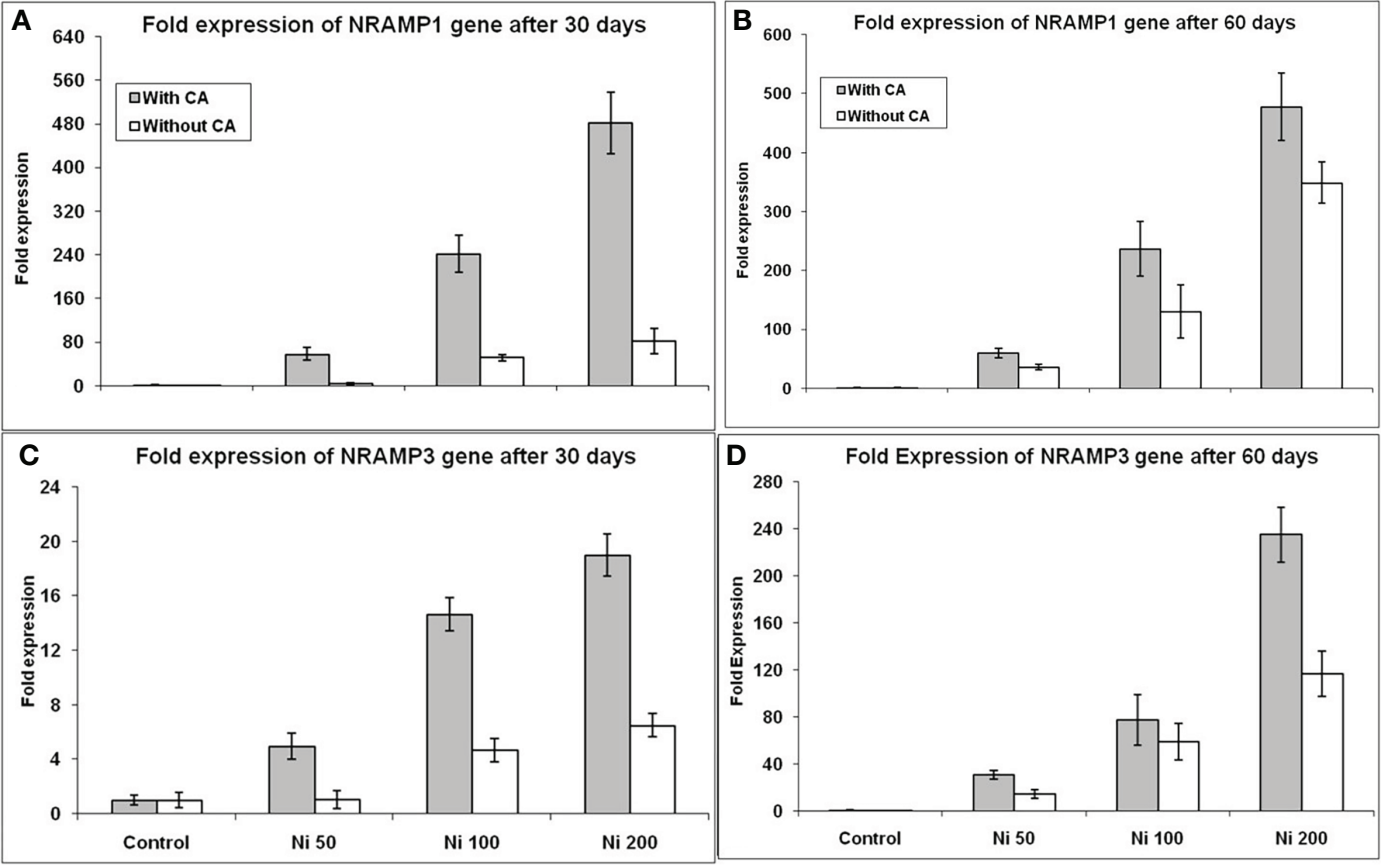
Gene expression profiling of *NRAMP* genes under Ni stress. *NRAMP1* expression on 30^th^ day **(A)**, *NRAMP1* expression on 60^th^ day **(B)**, *NRAMP3* expression on 30^th^ day **(C)**, and *NRAMP3* expression on 60^th^ day **(D)**, in sunflower plants. The bars designated with Ni 50, Ni 100, and Ni 200 represent Ni concentration at 50, 100, and 200ppm, respectively. “With CA” means these Ni-stressed plants are supplemented with 5 mM CA and vice versa for “Without CA”.

The increase in the expression of two metal transporters genes (*NRAMP1* and *NRAMP3*) was observed at both developmental stages. The expression of the *NRAMP1* gene was elevated up to 4, 51, and 81 folds under 50, 100, and 200ppm Ni-stress, respectively, in contrast to control and application of CA significantly enhanced the expression of *NRAMP1* gene further (**Figure 5A**) in 30 days old plants. After application of CA, the expression was elevated many fold, i.e. 7, 241 and 481 in 50, 100, and 200ppm Ni-stress, respectively. The same trend was observed for the expression of the *NRAMP1* gene in 60 day old sunflower plants (**Figure 5B**). Thus, 79, 141, 381% and 71, 136, 377% increase in *NRAMP1* expression was observed in 30 and 60 days old plants respectively treated with CA. The expression of the *NRAMP3* gene was elevated up to 1.05, 4, and 6 folds under 50, 100, and 200 ppm Ni-stress, respectively, in contrast to control and supplementation of CA had further elevated the expression of the *NRAMP3* gene upto 4.94, 14.65 and 19.02 folds respectively in 30 days old plants (**Figure 5C**). An exciting change was observed in the expression of the *NRAMP3* gene, as its expression was increased up to 30, 77, and 235 folds under 50, 100, and 200ppm Ni-stress, respectively, at the 60^th^ day in comparison to control (**Figure 5D**) in CA treated plants. In this way, the supplementation of Ni-stressed plants with CA increased the expression of both genes at both time intervals and 370, 213, 192% and 112, 31, 102% increase in *NRAMP3* expression was observed in 30 and 60 days old plants respectively treated with CA.

## 4 Discussion

A substantial quantity of Ni has been observed in both roots and shoots of sunflower plant in a dose-dependent manner at both development stages, while a greater tendency of retaining Ni was recorded in roots. The same phenomena in different plants were reported in Visioli et al. (2015) and Shahid et al. (2019) and in various plant species (like, maize, mung bean, and wheat), higher Ni content was found in roots than in shoots (Meychik et al., 2019; Abdelgawad et al., 2020). Our results are also in agreement with them and showed that the sunflower accumulated higher concentrations of Ni in shoots than roots. In the current study, the supplementation of CA on Ni-stressed plants resulted in an increased phytoextraction of Ni because organic acids increase the bioavailability of HMs (Mahmud et al., 2017). In our experiment, the sunflower accumulated high concentrations of Ni in shoots than in roots. Therefore, sunflower is not a hyperaccumulator for Ni but accumulated high concentrations of Ni in roots than shoots. In addition, the DTPA extractable Ni was raised with the application of CA at both time intervals (Aziz et al., 2007; Topcuoğlu, 2013).

Free proline, AsA, and protein act as antioxidants, metal chelators, and osmoprotectants. As, they are involved in different metabolic activities, may increase tolerance against HMs stress (Gill and Tuteja, 2010; Dawood, 2016; Nahar et al., 2016a, Nahar et al., 2016b; Jan et al., 2019; Mahata et al., 2019). In this study, the contents of proline, AsA, and protein were decreased in a dose-dependent manner and increased after the application of CA, indicating its role in combating Ni stress. The Phenolic compounds like flavonoids, phenolic acids, proanthocyanins, and tannins were reported to scavenge ROS in different plants (Wa et al., 2013). This research found that total phenolic contents were decreased under Ni-stress at both time intervals representing plant health is damaged due to Ni stress. Still, their levels were improved after applying CA, showing the positive roles of CA on plant health. In plants, the contents of photosynthetic pigments were decreased during metal stress because of lessened activities of Chl biosynthesis enzymes (Gill et al., 2015). Therefore, it could be stated that leaf chlorosis is due to the negative impact of HMs stress in plants. In our research, the concentrations were decreased in sunflower under Ni-stress and were noticeably improved by applying CA. Likewise, past research had reported that HMs toxicity was responsible for reducing Chl contents in different plants like barley, wheat, moss, and mustard plants (Choudhury and Panda, 2005; Ali et al., 2011; Ali et al., 2015; Gill et al., 2015). Furthermore, applications of various organic acids to a diverse plant group during different HMs stress restored the chlorophyll content (Ali et al., 2015; Zaheer et al., 2015).

ROS could act as secondary messengers by controlling the expression of various genes and proteins, but their higher levels can be toxic. The overproduction of H_2_O_2_ and MDA harms cell components like DNA, protein, carbohydrates, and lipids that eventually cause cell death in plants during stress (Nabi et al., 2019; Mahmud et al., 2019; Ni et al., 2019). In this study, the application of CA on Ni-stressed sunflower plants was found responsible for the decrease in the level of ROS and augmented the activities of antioxidants (SOD, POD, CAT, APX, DHAR, and GR). The SOD acts as a first line of defense during oxidative damage (Yadav et al., 2018; Mushke et al., 2019; Singh et al., 2019b) and scavenges superoxide and converts them into H_2_O_2_ that reactions carried out by POD could detoxify CAT and APX (Agar et al., 2019; Hasanuzzaman and Fotopoulos, 2019; Kiran and Prasad, 2019; Zhu et al., 2019; Akbari et al., 2020). Thus, upregulation of these enzymes is essential in combating HMs stress, and they are highly susceptible to the higher levels of Ni. In our study, the production of these enzymes was increased in the sunflower plants exposed to 50 ppm Ni-stress and decreased in the plants suffering from 100 and 200 ppm Ni-stress at 30^th^ and 60^th^ day, but with the application of CA, their levels were improved and ROS production was reduced which indicates their role in scavenging the ROS and protecting plants from oxidative damage. The activities of DHAR usually depends upon the production of ascorbic acid (Galli et al., 2019; Xiang et al., 2019). In this trial, the production AsA was reduced and concentration of DHAR was increased under 50ppm stress that is responsible for the reduction of ASA: DHA. The production of DHAR was also decreased under 100 and 200ppm Ni stress at both time intervals but it was improved by the supplementation of CA on Ni-stressed sunflower plants. The ascorbic acid could be oxidized to DHAR during abiotic stresses due to the scavenging activities of ROS (Mahmud et al., 2017), but reproduction of AsA is slow. Increased content of APX is also associated with the low production ascorbic acid in sunflower plants (Shehzad et al., 2020). By applying CA on Ni-stressed plants, increase in production of APX and DHAR was observed which eventually leads towards scavenging ROS and help plants to combat with oxidative damage in our study. Protein oxidation can be regulated by the production of glutathione reductase (Winterbourn, 2019). The GR content was increased in our study during 50 ppm Ni-stress that is important for the production of glutathione. The GR content was improved by applying CA at both time intervals in the present study.

The expression of metal transporter genes was higher in response to different HMs stress in hyperaccumulator plants (Mishra et al., 2017). Molecular studies have played a significant role in identifying the genes responsible for root to shoot translocation and the production of antioxidants (Hanikenne et al., 2008; Zhang and Evans, 2013; Peng et al., 2017; Maharajan et al., 2022) under HMs stress. Different transmembrane transporters like NRAMP are responsible for the uptake of HMs (Singh et al., 2019a). In some plant species, *NRAMP* transporter proteins have been reported in the transportation of HMs. For example, *TcNRAMP3/4* and *NcNRAMP1* were reported in Cd transportation in *Thlaspi caerulescens* and *Noccaea caerulescens* (Oomen et al., 2009; Milner et al., 2014). In *Thlaspi japonicum*, Ni and Cd are transported by *TjNRAMP4* (Mizuno et al., 2005). In the present study, the expression of *NRAMP1* and *NRAMP3* genes has been identified in Ni-stressed sunflower plants. The expression of these genes was increased at both time intervals with the application of CA because CA increases the bioavailability of Ni, which indicates their role in the transportation of Ni in the sunflower. The high production of antioxidants also represents that Ni is transported in high quantity from soil to the plants.

## Conclusions

The Ni toxicity in the soil is increasing day by day due to rapid urbanization and industrialization which results in stunted growth and reduced yield of essential food crops. To cope with this scenario, this study recommends growing plants like a sunflower in the Ni-polluted areas to reduce soil pollution, and applying organic acids, such as citric acid, to affected areas land would help maintain the plant health and enhance the rate of Ni uptake. We found that sunflower plant responded well regarding uptake of the Ni from the soil. So, the plant could be used for Ni phytoextraction, thus reducing soil toxicity. Furthermore, with the addition of CA, the uptake of Ni was further enhanced. The expression of Ni-responsive genes and variations in ROS production and antioxidants have suggested the importance of organic acids in reviving plant health under stress conditions. This study showed significant results in improving plant health under Ni stress. Use of sunflower may be effective to remove Ni from polluted soil, especially in industrial areas where land is severly polluted with metal.

## Data availability statement

The original contributions presented in the study are included in the article/supplementary material. Further inquiries can be directed to the corresponding authors.

## Author contributions

MIj and MA designed the study. MIj, MIq and HA conducted the experiment. HA, MZ, collected data, PR and MIj done with formal analysis of data. MIj, MIq, HA and SF drafted the initial manuscript., HA, DA, SF and HA helped in writing (review and editing). HA helped in data re-analysis, JI, and AA provided with literature review, language modification during revision process. MA and MIq done supervision and project administration Funding acquisition was undertaken by SF and KA. All authors contributed to the article and approved the submitted version.

## Acknowledgments

The authors would like to thank Princess Nourah bint Abdulrahman University Researchers Supporting Project number (PNURSP2022R292), Princess Nourah bint Abdulrahman University, Riyadh, Saudi Arabia. Moreover,authors are thankful to Higher Education Commission, Islamabad, Pakistan, for providing research facilities.

## Conflict of interest

The authors declare that the research was conducted in the absence of any commercial or financial relationships that could be construed as a potential conflict of interest.

## Publisher’s note

All claims expressed in this article are solely those of the authors and do not necessarily represent those of their affiliated organizations, or those of the publisher, the editors and the reviewers. Any product that may be evaluated in this article, or claim that may be made by its manufacturer, is not guaranteed or endorsed by the publisher.
